# A New Dental College

**Published:** 1865-09

**Authors:** 


### Editorial.

### A NEW DENTAL COLLEGE.

An effort is being made by the Profession in Michigan for the establishment of a Dental College in connection with Michigan University at Ann Arbor. There is a strong probability that the effort will be successful. Those connected with the Institution take a warm interest in the matter.

The plan proposed is to establish a chair of Operative Dentistry, and one of Mechanical Dentistry, and with them combine three of the Professorships from the present Medical Faculty, thus constituting a complete Dental Faculty. The Dental Course to be given after the close of the Medical Course. We think that much may be accomplished by some such arrangement. We have for a long time thought that the North-West should have a

Dental School, and this is certainly a very favorable opportunity for securing it. There have been many objections urged against connecting a Dental School with a Medical. Yet in this case such seem to be the conditions that few, if any objections exist so far as is now apparent. We hope the enterprise will succeed. It would certainly accomplish much for the Profession in the North-West.

